# Modeling neuromuscular diseases in zebrafish

**DOI:** 10.3389/fnmol.2022.1054573

**Published:** 2022-12-13

**Authors:** Jaskaran Singh, Shunmoogum A. Patten

**Affiliations:** ^1^INRS – Centre Armand Frappier Santé Biotechnologie, Laval, QC, Canada; ^2^Departement de Neurosciences, Université de Montréal, Montréal, QC, Canada; ^3^Centre d'Excellence en Recherche sur les Maladies Orphelines – Fondation Courtois (CERMO-FC), Université du Québec à Montréal (UQAM), Montréal, QC, Canada

**Keywords:** zebrafish, amyotrophic lateral sclerosis, neuromuscular deficits, Charcot Marie Tooth, spinal muscle atrophy

## Abstract

Neuromuscular diseases are a diverse group of conditions that affect the motor system and present some overlapping as well as distinct clinical manifestations. Although individually rare, the combined prevalence of NMDs is similar to Parkinson’s. Over the past decade, new genetic mutations have been discovered through whole exome/genome sequencing, but the pathogenesis of most NMDs remains largely unexplored. Little information on the molecular mechanism governing the progression and development of NMDs accounts for the continual failure of therapies in clinical trials. Different aspects of the diseases are typically investigated using different models from cells to animals. Zebrafish emerges as an excellent model for studying genetics and pathogenesis and for developing therapeutic interventions for most NMDs. In this review, we describe the generation of different zebrafish genetic models mimicking NMDs and how they are used for drug discovery and therapy development.

## Introduction

Neuromuscular diseases (NMDs) are a heterogeneous group of disorders that lead to muscle dysfunction and/or muscle wasting by affecting the muscles, motor neurons, peripheral nervous system or neuromuscular junction (NMJ). Most NMDs affecting the skeletal muscles lead to cardiac and respiratory dysfunctions (Alexandridis et al., 2022). The broad spectrum of NMDs differs in the age of onset, hereditary patterns, clinical symptoms and life expectancy.

Although individual NMDs are reported to be rare but as a group, the prevalence of NMDs is not very rare. Epidemiological assessment for the 24 most frequently occurring NMDs, reports their combined prevalence of 160/100,000 people (Deenen et al., 2015). The combined prevalence of NMDs is similar to the prevalence of Parkinson’s (100–300/100,000; Wirdefeldt et al., 2011) and is twice as high when compared to multiple sclerosis (80/100,000) in the European population. Some NMDs, such as Becker muscular dystrophy and Charcot Marie–Tooth disease, show an increasing trend in their prevalence rates since 1991 (Deenen et al., 2015). The lack of proper diagnosis and therapies has resulted in a 63% growth in the prevalence of NMDs among the population of the United Kingdom since 2000 (Carey et al., 2021).

Impaired neuromuscular transmission due to pre- and post-synaptic dysfunctions is the primary pathological process seen in most NMDs (Rubin, 2019). Pathological mechanisms of NMDs include, but are not limited to, presynaptic endoplasmic reticulum stress, disrupted mitochondrial bioenergetics, disrupted axonal transport, and impaired synaptic vesicle release in the presynapse and motor end plate defects, reduced acetylcholine receptors (AChRs), and blocked ion channels in the postsynapse (Chapman et al., 2013; Du et al., 2016; Boyd et al., 2017; Braz et al., 2021; Butti et al., 2021). Significant overlap between the symptoms of different NMDs makes it harder to diagnose them at an earlier stage and provide adequate care and treatment to patients. The late diagnosis makes it difficult to study the pathogenesis and genetics of most NMDs. This makes it difficult to design therapies for most NMDs, such as amyotrophic lateral sclerosis (ALS) and spinal muscular atrophy (SMA).

Advancements in next-generation sequencing technologies have helped to identify novel mutations and pathways in the pathogenesis of NMDs. Modeling these mutations in drosophila, mice and zebrafish has proven to be very useful in improving our understanding of pathological mechanisms underlying NMDs and for discovering potential therapeutics that are in clinical trials for some NMDs (Patten et al., 2017; Dowling et al., 2018; van Putten et al., 2020). In the past two decades, significant advancements in NMDs have been made by exploiting the attributes of the zebrafish that are complementary to the mammalian experimental systems. The zebrafish model possesses an anatomical and molecular architecture of the neuromuscular system similar to humans (Walogorsky et al., 2012; Chapman et al., 2013; Babin et al., 2014; Lipscomb et al., 2016; Butti et al., 2021; Koh et al., 2021). Zebrafish and human share 70% of the same genes, and more than 80% of disease proteins are conserved (Howe et al., 2013). Techniques for assessing neuronal and muscle activity *in vivo* in the zebrafish, such as recordings of synaptic currents in primary motoneurons and muscles, are well-established (Ali et al., 2000; Coutts et al., 2006; Patten and Ali, 2007, 2009; Butti et al., 2021), including optogenetics (del Bene and Wyart, 2012). Zebrafish is also a leading model for vertebrate genetics as it allows a wide range of genetic manipulations, including the latest genome engineering approaches (e.g., CRISPR/Cas9; Gao et al., 2017). Indeed, the innovative genetic manipulations in addition to behavioral assays make it an ideal model organism to mimic most human NMDs. Additionally, genetic mutations causing embryonic lethality in placental animals (as mice) can be easily analyzed in zebrafish embryos. The short generation period of the zebrafish makes it easy to trace the inheritance of NMD-associated genetic mutations over many generations and to analyze the penetrance and severity of the disease. Furthermore, zebrafish embryos/larvae are small in size and can be treated with chemical compounds in multi-well plates (96 or 384). Therefore, unlike mammalian models, they can serve as a *in vivo* vertebrate model for high-throughput drug screening for assessing drug efficacy and off-target effects in a whole-organism context. Noteworthy, several drugs identified in drug screening have comparable effects in zebrafish, mouse and human systems (MacRae and Peterson, 2015).

Here, we review genetic zebrafish models used to study NMDs. We will particularly discuss recent findings highlighting the strengths of zebrafish NMD models for providing novel insights into disease pathogenesis and drug discovery.

## Organization of human and zebrafish neuromuscular system

**Figure 1:**
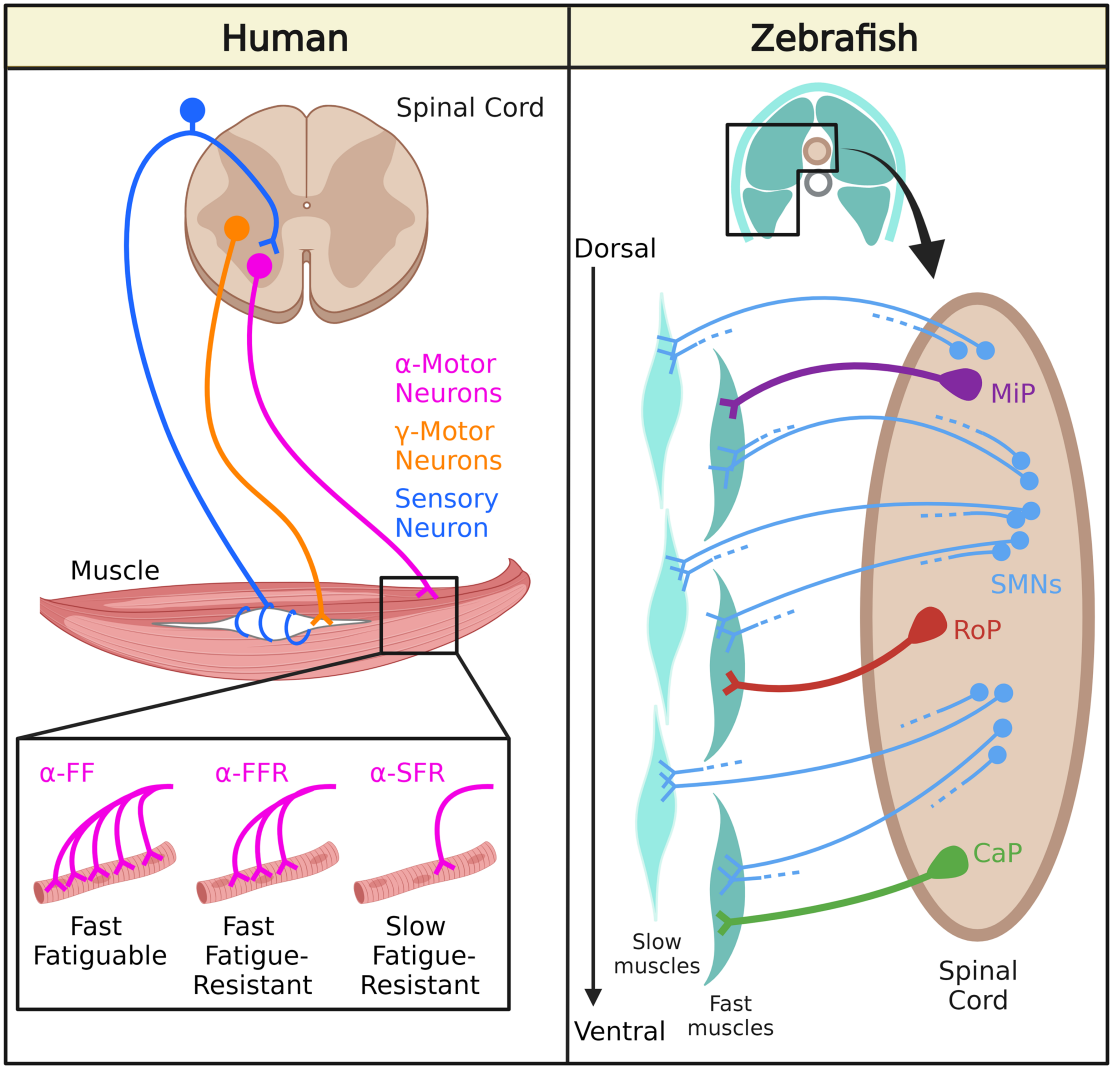
Neuromuscular system organization in human and zebrafish. In humans, γ-motor neurons innervate intrafusal muscle fibers while α-motor neurons innervate extrafusal muscle fibers: α-FF motor neurons densely innervate fast fatigable (FF) fibers, α-FFR motor neurons moderately innervate fast fatigue-resistant (FFR) fibers, and α-SFR motor neurons sparsely innervate slow fatigue-resistant (SFR) fibers. In zebrafish, secondary motor neurons (SMNs) innervate slow muscles and fast muscles. Middle primary motor neurons (MiP) innervate dorsal fast muscles, caudal primary motor neurons (CaP) innervate ventral fast muscles, and rostral primary motor (RoP) neurons innervate intermediate muscles.

Lower motor neurons in humans are classified according to their morphology and function as alpha motor neurons (α-MNs) and gamma motor neurons (γ-MNs; Figure 1). The α-MNs are the most abundant class of spinal motor neurons in humans and they innervate extrafusal muscle fibers responsible for muscle contraction and skeletal movements. They can be subdivided into three categories based on the contractile properties of their motor unit’s target muscle fibers: (1) Slow-twitch fatigue resistant (S) with smaller cell bodies and axons; (2) Fast-twitch fatigue resistant (FR); and (3) Fast-twitch fatigable (FF) with large cell bodies and axons (Burke et al., 1973). The γ-MNs innervate intrafusal fibers of the muscle spindle, and regulate sensitivity of the muscle spindle in response to stretch.

Zebrafish spinal MNs are classified into primary motor neurons (PMNs) and secondary motor neurons (SMNs) based on their innervation of target muscle fibers (Eisen et al., 1986; Myers et al., 1986; Figure 1). PMNs can be further categorized into three subtypes: (1) caudal primary (CaP) motor neurons (CaP)- innervating the ventral trunk musculature; (2) middle primary (MiP) motor neurons- innervating dorsal trunk musculature, and (3) rostral primary (RoP) motor neurons- innervating muscle fibers in between the ventral and dorsal trunk musculature (Moreno and Ribera, 2009). On the other hand, SMNs are located more ventrally in the motor column. During the first day of development, PMNs undergo axogenesis and are relatively larger (Lewis and Eisen, 2003). SMNs appear later and they are relatively smaller and more numerous compared to PMNs. They also have thinner axons than PMNs. The dorso-ventrally-projecting SMNs (dvS) innervate both dorsal and ventral musculature, whereas the ventrally-projecting SMNs (*vS*) have ventrally restricted arborization fields. These two types of SMNs exhibit extensive branching from the main axonal branch deep into the muscle fibers, indicating that they preferentially activate fast muscles. The dorsally-projecting SMNs innervate the dorsal musculature and activate fast skeletal muscles. SMNs are similar to human α-MNs, but zebrafish PMNs do not have a human equivalent, and human γ-MNs do not exist in zebrafish.

Axial motor structures consisting of a rostro-caudal series of myomeres are conserved in most anamniotic vertebrates, including zebrafish (Fetcho, 1992). There are also many molecular and anatomical similarities between skeletal muscle fibers in zebrafish and mammalian muscle fibers, such as the components of the dystrophin-associated glycoprotein complex, which is a part of the excitation-contraction coupling machinery and contractile apparatus (Gibbs et al., 2013). Two types of muscle fibers make up the myomeric musculature in zebrafish. The first class of fibers is slow fibers which form a superficial monolayer on the myotome. These fibers rely on oxidative phosphorylation and are more resistant to fatigue. The other class of fibers is called fast fibers, which make up the deep portion of the myotome and are more fatigable as they rely on anaerobic glycolysis for ATP generation (Welsh et al., 2009). Each fast fiber is innervated by one PMN and four SMNs, whereas each slow fiber is innervated only by several SMNs (Westerfield et al., 1986). Fast fibers are activated during fast swimming or escape movements, and slow fibers are generally used during slow swimming in zebrafish (Fetcho, 1992).

## Spinal muscular atrophy

Spinal muscular atrophy (SMA) is an autosomal recessive neuromuscular disorder (Lunn and Wang, 2008). The disease is characterized by the loss of lower motor neurons that results in muscular atrophy and paralysis, which ultimately leads to death, mostly by respiratory failure (Lunn and Wang, 2008; Mercuri et al., 2012). The pathology of the disease is associated with homozygous deletion or mutation of the ubiquitously expressed *SMN1* gene present on chromosome 5q13, resulting in reduced levels of SMN protein (Francis et al., 1993; Kleyn et al., 1993). Additionally, humans possess a paralog of the *SMN1* gene, “*SMN2*” that, due to a nucleotide change in exon 7 (C to T), produces low levels of full-length SMN protein (10% of *SMN1* levels; Lorson et al., 1999; Monani et al., 1999). The copy number of the *SMN2* gene in SMA patients is inversely proportional to the severity of the disease (Taylor et al., 1998; Monani et al., 2000). SMA has been clinically subdivided into four categories based on the age of onset and motor functioning (Munsat and Davies, 1992). Type 1 SMA or Werdning–Hoffman disease is the most severe type of SMA that occurs before 6 months of age and results in death within the first 2 years of life. Hypotonia, muscle weakness and poor motor development are the characteristic features of SMA type I. Scoliosis, along with other skeletal abnormalities, has been reported in SMA I and the affected children are not able to sit or stand. In addition, bulbar denervation is responsible for poor sucking and swallowing abilities in children suffering from SMA I. SMA type II is of intermediate severity and occurs between the ages of 7 and 18 months (Edens et al., 2015). Children suffering from SMA type II can sit but not stand or walk independently. Along with the loss of motor skills, children exhibit proximal muscle weakness and postural tremor of the fingers (Elsheikh et al., 2020). SMA type III or Kugelberg–Welander disease usually develops after the age of 18 months, with children being able to walk independently (Edens et al., 2015). Proximal muscle weakness is generally seen in type III SMA with difficulty in running and climbing stairs (Elsheikh et al., 2020). Postural tremor of the fingers is also reported and loss of motor skills may occur with time (Elsheikh et al., 2020). Adult-onset is a characteristic feature of SMA type IV. Cardiac and respiratory functions are seen as normal in SMA type IV but patients experience mild muscle weakness and hand tremors (Elsheikh et al., 2020).

Different zebrafish models have been created to study the pathogenesis or SMA (Table 1). One of the two proposed hypotheses for the selective vulnerability of motor neurons in SMA is the reduction in the biogenesis of snRNP (Pellizzoni et al., 2002). The catalytic activity of SMN has been reported to assemble Sm proteins into heptameric rings around snRNA to form a snRNP complex (Chari et al., 2008). Hence, reduced SMN levels affect the snRNP complex assembly, thereby affecting the splicing of transcripts essential for motor neurons (Winkler et al., 2005; Chari et al., 2009; See et al., 2014). The localization of mCherry-SMN (~60 kDa) is seen to be lower in the nucleus and higher in the cytoplasm of SH-SY5Y neuroblastoma cells, despite 90–110 kDa being the size limit of the proteins to freely diffuse into the nucleus (Wang and Brattain, 2007; Koh et al., 2021). Importers such as snurportin and importin β translocate the SMN protein to the nucleus as a part of the snRNP complex (Narayanan et al., 2002), explaining the weak expression of mCherry-SMN (~60 kDa) in the nucleus. Confocal-Fluorescence Correlation Spectroscopy (FCS) measurements in the motor neurons of 2 days post fertilization (dpf) zebrafish revealed the population of SMN proteins can be separated into a fast-diffusing and slow-diffusing component (Koh et al., 2021). It has been reported that the SMN protein oligomerizes (Pellizzoni et al., 2002) and thus associates with Gemin 2–8 and UNR-interacting Protein (UNRIP) to form an SMN complex (Cauchi, 2010). The fast and slow components represent the monomeric and oligomeric forms of SMN protein, respectively, both of which diffuse into the axons instead of being actively transported (Koh et al., 2021). Additionally, oligomerized SMN protein interacts with other protein complexes involved in RNA metabolism, translation and transcription. Therefore, it is not clear whether the oligomerized SMN observed by FCS were part of SMN complexes or other protein complexes in the motor neurons of the 2 dpf zebrafish. Exons 6 and 7 of *SMN1* are important for axonal localization and dimerization of the SMN protein (Lorson et al., 1998). An *hb9:eGFP-Smn*Δex6,7 plasmid was generated and inserted into zebrafish to check for SMN localization and dynamics disturbances in 2 dpf zebrafish motor neurons (Koh et al., 2021). The slow component that represents the oligomeric form of the SMN protein was no longer present in the cytoplasm of the cell body of motor neurons of *hb9:eGFP-Smn*Δex6,7 fishes but was strongly expressed in the nucleus. Therefore, it shows that SMN lacking exons 6 and 7 loses its ability to self-oligomerize, which will likely inhibit its ability to become part of the SMN complex although it may still become part of other protein complexes.

**Table 1:** Zebrafish SMA models and their distinct phenotypes.

**Zebrafish SMA models**	**Characteristic phenotypes**	**Method used to create the line**
*smn* morphant	• Truncation of caudal primary motor neurons (CaP MN)	Morpholino knockdown of *smn* gene
	• Extensive branching of CaP MNs	
	• Reduced mitochondrial ATP biogenesis	
	• Reduced mitochondrial respiration rate	
*pgk1* morphant	• Truncation of CaP MNs	Morpholino knockdown of *pgk1* gene
	• Abnormal branching of MN axons	
SmnA6T^ind27^	• Significant reduction in MN number	CRISPR-Cas9-mediated homology-directed repair to induce an A6T substitution and a 27 bp indel within exon 7 of the *smn* gene
	• Loss of regularly shaped polygonal muscle fibers	
	• Presence of small myofibers interspersed between hypertrophic myofibers with enlarged cross-sectional areas	
	• Reduced motor activity	
*Smn-*mir	• Short CaP MNs axons	miRNA-based heritable genetic knockdown of the *smn* gene
	• Abnormal branching of CaP MNs	
	• Pathfinding defects	
	• Motor impairment	
	• Weight loss, abnormal swimming behavior, scoliosis	
*chodl−/−; mnx1:*EGFP	• Shorter motor axons	CRISPR mediated 4 bp deletion in the open reading frame near the start codon of *chodl gene*
	• Pathfinding defects	
	• Reduced innervation of myotomes	
	• Impaired axonal branching	
	• Abnormal touch evoked response	
*exosc8* morphant	• Increased AU-rich myelin basic protein	Morpholino knockdown of *exosc8* gene
	• Abnormal hindbrain motor neuron development	

An interesting fact is that different motor neuron pools exhibit differences in vulnerability to degeneration in SMA depending on their different anatomical muscle projections (Murray et al., 2008; Ling et al., 2012). In the SMA mouse model, it has been reported that among three different hind limb muscles, the extensor digitorum longus (EDL) and intermediate gastrocnemius (GS) muscles are disease resistant, while the tibialis anterior (TA) muscle is vulnerable to degeneration (Murray et al., 2008). Transcriptomic analysis of the MN pools projecting to the above three muscles in 14-day postnatal mice was performed. A comparative gene expression profile revealed the enrichment of mitochondrial genes in the MN pools innervating the disease-resistant extensor digitorum longus (EDL) and intermediate gastrocnemius (GS) muscles (Boyd et al., 2017). The argument that can back up this fact is that ATP generation is critical for tissues with high metabolic demands, such as motor neurons, to support basic cellular functions and maintain action potentials throughout long axons. A hindrance in the bioenergetic pathway may therefore be the reason for the selective degeneration of MNs in most neuromuscular disorders. In a *smn* morpholino knockdown zebrafish model, it has been found that ATP synthase subunit alpha (ATP5A), which produces ATP from ADP, is significantly reduced, replicating the results of mitochondrial bioenergetics in the SMA mouse model (Boyd et al., 2017). Additionally, Seahorse XF analyzer measurements that measure mitochondrial respiration revealed a very low respiration rate in the *smn* morphant fish. Enhancement of mitochondrial biogenesis in *smn* morphant fish by necdin, promoter of neuronal mitochondrial biogenesis (Hasegawa et al., 2016), rescues the axonal motor neuron phenotype (Boyd et al., 2017). Phosphoglycerate kinase 1 (PGK1) is an enzyme present in the axons and growth cones of MNs, suggesting it has a glycolytic role in local ATP generation. Concentration-dependent morpholino knockdown of *pgk1* in zebrafish produces truncation and abnormal branching in MN axons, which mimics the SMA phenotype. Overexpression of *pgk1* mRNA in *pgk1* morphants rescues the motor neuron phenotype (Boyd et al., 2017). These results therefore suggest that the alteration of mitochondrial bioenergetic pathways can induce SMA. Therefore, bioenergetic therapies could be a potential approach for treating SMA and other NMDs.

Most zebrafish models mimic the severe forms of SMA *via* morpholino-mediated knockdown of their only *smn* gene, which makes it difficult to study the mild and intermediate forms of SMA due to the early lethality of the embryos. Using CRISPR-Cas9-mediated homology-directed repair (HDR) in zebrafish, an A6T substitution and a 27 bp indel within exon 7 of the *smn* gene (SmnA6T^ind27^) was introduced to more closely mimic the intermediate form of hSMN2-dependent SMA (Tay et al., 2021). The SmnA6T^ind27^ protein persisted until 5 dpf and started to decline strongly at 12 and 42 dpf. The average survival rate of SmnA6T*^ind27^* mutants was 32 dpf compared to 20 dpf for smnΔ7*^1–14^* mutants (14 bp deletion in a highly conserved region between smn and hSMN1). No significant difference was observed in body length until shortly before death, which is a similar scenario seen in SMA patients (Arnold et al., 2015). At 36 dpf a significant reduction in SV2 labeling marking for MN number and the presence of small myofibers interspersed between hypertrophic myofibers with enlarged cross-sectional areas were observed, which are key pathological features in the biopsies of SMA patients (Pérez-García et al., 2017). SmnA6T*^ind27^* developed normally and survived longer than other zebrafish SMA models, thus mimicking the intermediate form of SMA.

Furthermore, a zebrafish model with heritable genetic knockdown of the SMN gene based on miRNAs has been developed, which mimics the various forms of the disease with varying potencies (Giacomotto et al., 2015). The miRNA-containing plasmids with different backbones targeting the 3′-UTR were co-injected with transposase mRNA to promote DNA integration. A strong reduction of SMN protein was observed in F1 zebrafish transgenic larvae at 72 h post fertilization (hpf) and 6 dpf suggesting a stable and efficient heritable knockdown of the *smn* gene *via* a miRNA-based approach. F1 transgenic fish showed a characteristic SMA motor neuron phenotype with short axons and abnormal branching. F2 transgenic lines with one of the DNA constructs (UBI:miRsmn1-1#5) exhibited a severe form of SMA with a 90% reduction in SMA protein at 52 hpf and a maximum survival age of 11 dpf. F2 transgenic larvae also exhibited poor motor functions with reduced swimming distance and speed, as seen in SMA cases. Injections of hsa-SMN1 mRNA into F2 UBI:miRsmn1-1#5 embryos in a dose-dependent manner (200 and 400 pg) rescued the motor neuron phenotype, confirming the specific knockdown of *smn* in transgenic fish. This zebrafish model gives an advantage in that partial knockdown of a target gene is less likely to induce a molecular compensation, which can be seen in full knockdown approaches. Also, this model can mimic the different forms of SMA that can survive until adulthood; therefore, they can be easily used for high throughput drug screening assays for SMA, which is a major limitation in other animal models.

Despite all of the research done on smn, it is still unclear why low levels of this protein selectively degenerate motor neurons. Some non-canonical pathways have come to light that may shed light on this question. *Chodl* encodes for the chondrolectin protein and it is highly expressed in the spinal motor neurons of mice and humans (Bäumer et al., 2009; Enjin et al., 2010; Wootz et al., 2010). Reduction of *Chodl-001* expression before the onset of symptoms in SMA has been reported in an SMA mouse model (Sleigh et al., 2014). Knockdown of *chodl* in zebrafish results in shorter motor neuron axons and reduced muscle innervation at later developmental stages (Zhong et al., 2012), which is a similar phenotype seen in *smn* zebrafish knockdown models (Carrel et al., 2006; Liu et al., 2011). Co-injection of *Chodl* mRNA with *smn* morpholino rescues the motor neuron phenotype in zebrafish with improved morphology, growth and pathfinding (Sleigh et al., 2014). Because *chodl* results in shorter CaP MNs in zebrafish, different synapse-stabilization compounds have been tested in zebrafish *chodl−/−* mutant with a GFP reporter expressed under the motoneuron promoter *mnx1 (chodl−/−; mnx1:EGFP)*. An automated *in vivo* screening of 982 small molecules was performed *in chodl−/−; mnx1:EGFP* mutant zebrafish line, and the length of CaP MNs was assessed as a parameter for the potential rescue of the SMA phenotype (Oprişoreanu et al., 2021). Out of 982 small molecules screened, four compounds significantly increased the CaP MN length, which were: (1) dipyridamole, a non-selective phosphodiesterase and adenosine uptake inhibitor; (2) IOX1, a broad-spectrum inhibitor of 2OG oxygenases; (3) MG132, a proteasome inhibitor and; (4) apicidin, a histone deacetylase inhibitor. Furthermore, NMJ morphology analysis revealed that dipyridamole and IOX1 could rescue pre-synaptic defects in *chodl−/−* mutant zebrafish at the horizontal myoseptum synaptic site.

EXOSC8 is an essential protein of the exosome core, that is responsible for the degradation of AU-rich element containing mRNAs (Chen et al., 2001; Makino et al., 2013). EXOSC8 mutation in a Hungarian family is reported to have an overlapping phenotype of hypomyelination, cerebellar hypoplasia and SMA (Boczonadi et al., 2014). MO knockdown of the *exosc8* gene in zebrafish results in abnormal hindbrain motor neuron development with an increase in AU-rich myelin basic protein (MBP) mRNAs (Boczonadi et al., 2014). Thus, studying the subset of genes that presymtomatically produce SMA phenotypes along with the *smn* gene phenotype can give insights into the molecular mechanisms working behind the selective degeneration of lower motor neurons in SMA.

Disruption in ubiquitin homeostasis is reported as a key feature in SMA pathogenesis (Aghamaleky Sarvestany et al., 2014; Wishart et al., 2014). Ubiquitin-like modifier activating enzyme 1 (UBA1) is an enzyme that catalyzes the first step in ubiquitination, and reduced levels of UBA1 are observed in SMA mice (Wishart et al., 2014). Furthermore, suppression of *Uba1* is sufficient to recapitulate the SMA-like phenotype in zebrafish. Co-injection of human UBA1 mRNA along with *smn* morpholinos in zebrafish eggs ameliorates motor axon morphological defects in *smn* knockdown zebrafish. Additionally, swim path analysis of UBA1 mRNA coinjected fish reports the restoration of motor capacity in *smn* knockdown zebrafish (Powis et al., 2016). The results were then further analyzed using a gene therapy approach in an SMA mouse model. Intravenous injection of AAV9-UBA1 in SMA mice resulted in improved motor functions, the rescue of neuromuscular junction morphology and increased spinal motor neuron survival (Powis et al., 2016). These findings demonstrate the potential of the systematic therapeutic approach in SMA *via* targeting ubiquitin pathways.

## Duchenne muscular dystrophy

Duchenne muscular dystrophy (DMD) is the most common neuromuscular disorder, which results in severe and progressive loss of heart and skeletal muscles, consequently leading to death due to heart or respiratory failure (Hoffman et al., 1987; Wu et al., 2014). DMD affects ~1 in 3,500–5,000 male births and presents its motor symptoms during early childhood (Emery, 1991; Crone and Mah, 2018). It progresses rapidly, leaving most affected individuals in a wheelchair during their teenage years and usually becoming fatal during the third or fourth decade of life (Emery, 1991). This X-linked disorder occurs due to a loss-of-function mutation in the DMD gene that encodes for the dystrophin protein (Monaco et al., 1986; Hoffman et al., 1987). Dystrophin is a key component of the dystrophin-associated protein complex (DAPC) in the sarcolemma of muscle cells (Ervasti et al., 1990; Yoshida and Ozawa, 1990). Dystrophin and DAPC have been seen to play a role in regulating nitric oxide production and Ca^2+^ entry (Bodensteiner and Engel, 1978; Brenman et al., 1995; Allen et al., 2016). Loss of DAPC and dystrophin makes muscle cell membranes susceptible to contraction induced damage (Petrof et al., 1993; Dellorusso et al., 2001).

Corticosteroids are the current standard treatment for DMD, which delays muscle dysfunction but exhibits serious side effects such as weight gain, osteoporosis and a cushingoid appearance (Bushby et al., 2010; Kinnett et al., 2015). Therefore, some small molecules have been identified to modulate pathological mechanisms downstream of dystrophin, resulting in ameliorating DMD phenotypes. Zebrafish are a convenient model for testing a large number of small molecules. Zebrafish eggs can readily absorb the small molecules and the larvae can be further tested for muscular structures using polarized light birefringence techniques (Kawahara et al., 2011; Berger et al., 2012). The mutant *dmd* zebrafish line *sapje,* having a nonsense mutation in exon 4, mimics many aspects of human DMD pathology, including skeletal muscle fibrosis and inflammation (Bassett and Currie, 2004; Berger et al., 2010).

Histone deacetylase inhibitors (HDACi) are a class of epigenetic small molecules that have shown promising effects on DMD mutant animal models and in clinical trials (Minetti et al., 2006; Johnson et al., 2013; Bettica et al., 2016). Givinostat, an HDACi, has shown some potential results in clinical trials. Among the class of HDACs, HDAC8 is reported to have a unique structure as it lacks the C-terminal (aa 50–111) protein-binding domain and possesses a flexible L1 loop in the proximity of the active site, which allows it to accommodate different substrates (Somoza et al., 2004). HDAC8 upregulation has been observed during the differentiation of human DMD myoblast cells (Spreafico et al., 2021). The specific inhibition of HDAC8 *via* PCI34051 results in a significant increase of the fusion index, suggesting the role of HDAC8 in DMD pathology (Spreafico et al., 2021). HDAC8 expression was also observed *in vivo* by using zebrafish as a model organism. Increased expression of HDAC8 mRNA and protein is found at 48 hpf and 72 hpf in *dmd*-MO injected zebrafish embryos (Spreafico et al., 2021). Givinostat and PCI3405, a pan-HDACi and HDAC8i, respectively, partially rescue the muscle lesion phenotype presented at 72 hpf in *dmd*-MO injected zebrafish larvae (Spreafico et al., 2021). Inflammation, a secondary effect of dystrophin deficiency, is rescued to a greater extent with PCI than with Givinostat, which points out the potential of specific HDAC-targeting therapies compared to pan-HDAC inhibition. Furthermore, acetylome analysis in PCI-treated zebrafish has found α-tubulin as a target of HDAC8. Reduction of acetylated tubulin levels is found in *dmd*-MO injected zebrafish embryos, which are then further rescued by PCI treatment (Spreafico et al., 2021), illustrating the role of HDAC in modulating the cytoskeleton architecture *via* acetylation of its targets.

**Figure 2:**
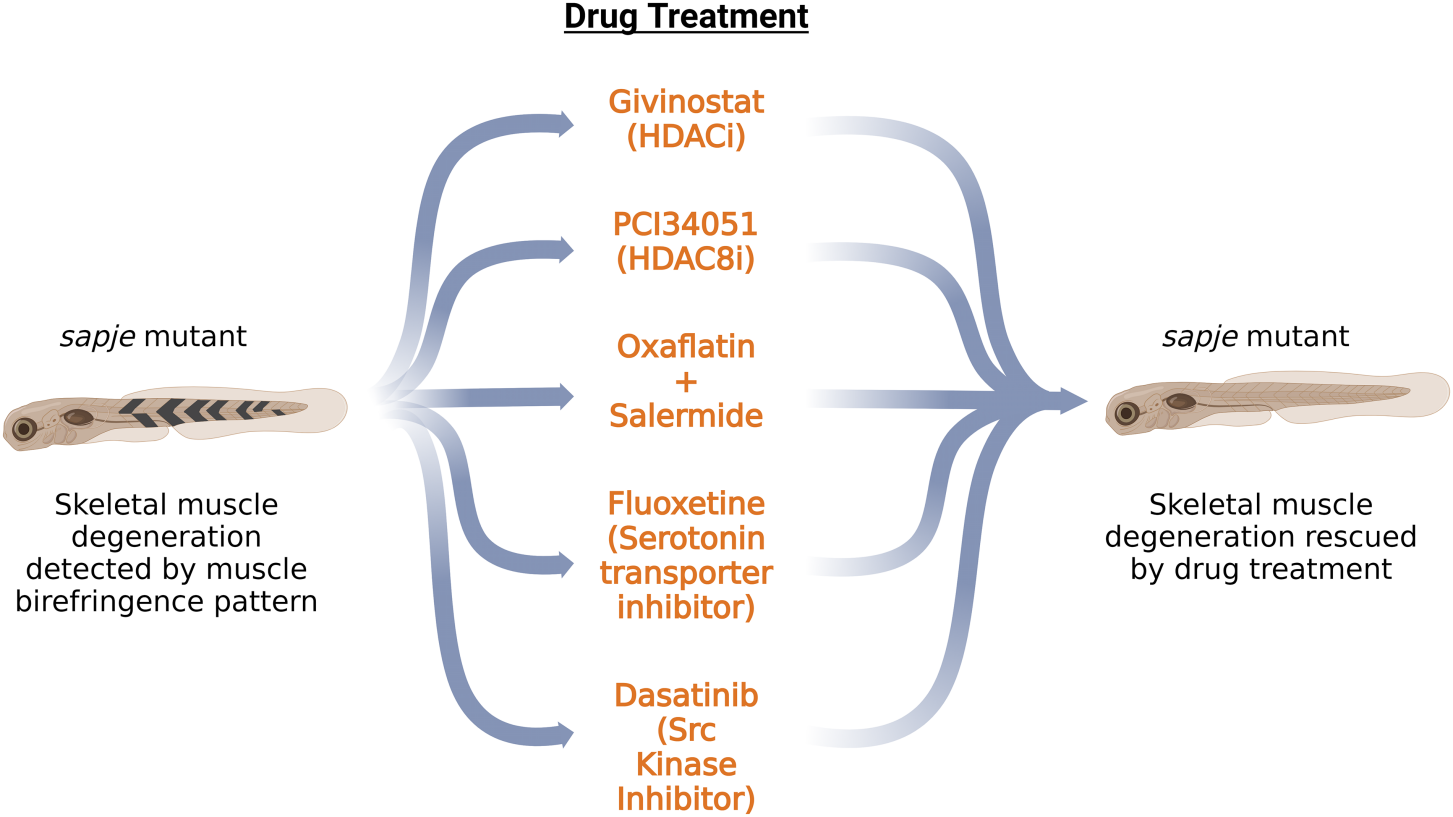
Schematic summary of the potential drugs tested on *sapje* zebrafish DMD model. Application of these drugs on *dmd* mutant zebrafish rescued the skeletal muscle degeneration as observed by the muscle birefringence pattern. HDACi: Histone Deacetylase inhibitor; HDAC8i: Histone Deacetylase 8 inhibitor.

As gene therapy approaches have not been successful in restoring the most common *dmd* mutation to restore the missing sequence, many small molecules and drugs are being tested to improve or alleviate the symptoms of the disease (Figure 2). Apart from HDACi, another large class of epigenetic small molecules is yet to be explored in the context of DMD pathology. Farr et al. tested epigenetic small molecules from the Cayman Chemical Epigenetics Screening Library on *dmd* mutant zebrafish line *sapje*. They developed a grid system containing 403 chemical pools, which would allow testing of each compound in the library in combination with every other library compound at least once (Farr et al., 2020). Their method was validated against TSA-containing chemical pools which rescued the skeletal muscle phenotype in *sapje* zebrafish. Further analysis narrowed the candidate pools to a specific row of chemical pools in the plate that significantly increased the brightness level of the muscle birefringence, pointing to their beneficial effect on the *dmd* mutant fish. In contrast, individual testing of each compound from the pool did not show any significant effect, highlighting the use of combinations of drugs for future therapeutic approaches. Oxaflatin in combination with salermide significantly improved the *dmd* mutant muscle birefringence, without rescuing dystrophin levels (Farr et al., 2020). Further studies would be needed to determine if oxaflatin and salermide work in an additive or synergistic manner.

Waugh et al., have performed a large drug screen of 640 FDA-approved compounds on the *sapje* zebrafish line. Of the 640 compounds tested, six positive hits were obtained, as determined by the muscle birefringence pattern. The drug candidate hits belonged to a broad class of monoamine agonists such as pergolide, ergotamine and fluoxetine (Waugh et al., 2014). Testing of additional monoamine agonists pointed to serotonin as a potential candidate for preventing the development of abnormal birefringence in *sapje* zebrafish embryos. These data draw attention to the serotonin pathway in modulating DMD pathology. Morpholino knockdown of *slc6a4b*, a serotonin transporter, rescues the birefringence pattern in *sapje* zebrafish embryos (Waugh et al., 2014). This validates the action of fluoxetine, which prevents the serotonin reuptake by inhibiting the serotonin transporter. Additionally, fluoxetine works independently of dystrophin expression and restores internal muscle architecture. As serotonin is a regulator of vascular tone, it might be possible that the regulation of bloodflow to the muscles might play a role in DMD pathogenesis.

β-dystroglycan associates with dystrophin and other proteins to form the dystrophin glycoprotein complex (DGC; Kaufmann, 2003). The tyrosine-phosphorylation of this protein controls the association of dystrophin with its binding partners and also acts as a signal for the degradation of β-dystroglycan (James et al., 2000; Ilsley et al., 2001; Sotgia et al., 2003). In the dystrophin-deficient *mdx* mouse model, preventing the phosphorylation of β-dystroglycan alleviates the dystrophic phenotype (Miller et al., 2012). Additionally, proteasomal inhibition in *mdx-*mice restores dystrophin glycoprotein complex (DGC) components. This leads to the argument that the loss of dystrophin triggers the increased phosphorylation of β-dystroglycan on tyrosine which then triggers proteasomal degradation of β-dystroglycan along with DGC at the sarcolemma leading to dystrophic phenotypes (Assereto et al., 2006; Minetti et al., 2006). The levels of β-dystroglycan were examined in the *sapje* zebrafish model of DMD. Initially, the levels of non-phosphorylated β-dystroglycan were significantly lower compared to WT siblings, but by 5 dpf, both the non-phosphorylated and phosphorylated forms are seen to be reduced when compared with control siblings (Lipscomb et al., 2016). It is possible that before 5 dpf, a dynamic equilibrium was established as non-phosphorylated β-dystroglycan was phosphorylated in order to achieve a required amount of phosphorylated β-dystroglycan, and that at 5 dpf, the non-phosphorylated pool was depleted. Additionally, western blot analysis of dystrophin-deficient *sapje* zebrafish shows some high molecular weight β-dystroglycan bands around 53 and 63 kDa deviating from normal 43 kDa β-dystroglycan protein (Lipscomb et al., 2016). A pull-down assay of myoblast lysates with glutathione-S-transferase (GST)-MultiDsk, a protein comprising of three repeated ubiquitin binding domains fused to GST, was performed. An antibody specific to tyrosine-phosphorylated β-dystroglycan was able to recognize the high molecular weight species, while an antibody specific to non-phosphorylated β-dystroglycan failed to detect the higher molecular weight species. This indicates the presence of ubiquitin moieties on phosphorylated β-dystroglycan (Lipscomb et al., 2016).

One of the kinases that phosphorylate tyrosine on β-dystroglycan is Src kinase whose kinase activity is the initial step in the degradative cascade (Lipscomb et al., 2016). Dasatinib, an Src kinase inhibitor, restores the muscle birefringence pattern in dystrophic deficient *sapje* zebrafish (Lipscomb et al., 2016). Furthermore, it restores muscle function, as indicated by the improved swimming activity of *sapje* zebrafish larvae at 5 dpf. Dasatinib was ineffective in restoring the muscle architecture in dystroglycan null zebrafish, showing that dasatinib’s acts by rescuing the excessive phosphorylation of dystroglycan (Lipscomb et al., 2016). Therefore, restoring the levels of β-dystroglycan in the absence of dystrophin *via* tyrosine kinase inhibitors, ubiquitination inhibitors and inhibitors of proteasomal degradation could pave a path for future therapeutic approaches.

Guillaume Benjamin Amand Duchenne, a French neurologist who described DMD in 1861, proposed the idea of electric stimulation as a potential therapy for dystrophin-deficient muscles (Duchenne et al., 1871; Barnard et al., 1986). Elisabeth et al. performed a longitudinal study on *sapje* zebrafish DMD mutant with four paradigms of neuromuscular electric stimulation (NMES), ranging from low frequency and high voltage to high frequency and low voltage (Kilroy et al., 2022). The four NMES paradigms were named as Power NMES (pNMES), Strength NMES (sNMES), Hypertrophy NMES (hNMES) and Endurance NMES (eNMES) with pNMES being the low frequency and high voltage stimulation and eNMES being high frequency and low voltage stimulation. Three sessions of each NMES of 1 min each day were performed on 2, 3, 4 dpf WT and *dmd* mutant zebrafish larvae, followed by a recovery period of 4 days. The muscle birefringence pattern was then analyzed, which showed a significant increase in birefringence for *dmd* mutants following eNMES and pNMES. Muscle structure analyzed *via* phalloidin staining showed fewer degeneration of fibers in *dmd* mutants following eNMES and pNMES. Additionally, all NMES paradigms except pNMEs showed an increased number or length of NMJs in dmd mutant fish (Kilroy et al., 2022). Furthermore, the assessment of motor behavior revealed an increase in swimming distance and velocity in *dmd* mutants following eNMES compared to control *dmd* mutants, providing evidence of improved muscular function following an improved muscular structure (Kilroy et al., 2022). Sarcomere length impacts muscle function (Moo and Herzog, 2018), therefore, the length of sarcomere was also assessed in 8 dpf *dmd* mutants following eNMES and found to be significantly increased compared to control fish. Heme oxygenase (HO) is an anti-inflammatory and an antioxidant agent, and it has already been implicated as a potential therapeutic treatment for both zebrafish and mouse *dmd* models (Kawahara et al., 2014; Chan et al., 2016). Analysis of RNA sequencing data revealed upregulation of HO in *dmd* mutant zebrafish larvae following eNMES training. Injection of *hmox1a* morpholino at 1 dpf in *dmd* mutants following eNMES protocol showed no improvement in muscle organization and fiber detachment (Kilroy et al., 2022). This suggests that HO is necessary for eNMES-mediated recovery.

## Charcot Marie Tooth disease

Charcot–Marie–Tooth disease (CMT) is a group of inherited peripheral neuropathies affecting sensory and motor neurons. It is principally subdivided into CMT1 and CMT2. CMT1 is associated with demyelination of the axons resulting in reduced nerve conduction velocities. CMT2 is linked with axonal degeneration which could be due to disturbed mitochondrial dynamics, axonal transport or protein homeostasis. CMT is a progressive condition that usually displays symptoms between 5 and 15 years of age but can also occur in midlife or later ages. People suffering from CMT experience leg, ankle, and foot muscle weakness and a highly arched or flat foot presentation.

Mitofusin 2 (MFN2) is a mitochondrial membrane protein that regulates mitochondrial fission and fusion events (Santel and Fuller, 2001). MFN2 mutation is reported as the leading cause of CMT type 2A in patients (Züchner et al., 2004). Disturbances in mitochondrial dynamics have been reported in many neurodegenerative disorders (Yang et al., 2021), which may also account for axonal degeneration in CMT2. MFN2 protein is highly conserved between humans and zebrafish with 82% amino acid identity and a conserved GTPase domain, transmembrane domain and two heptad coiled-coil domains (Chapman et al., 2013). A T > A mutation in exon8 of zebrafish MFN2 introduces a stop codon at leucine 285 (MFN2^L285X^) after the GTPase domain rendering the MFN2 protein non-functional (Chapman et al., 2013). Primary neuronal cultures derived from ENU-induced 24 hpf MFN2^L285X^ mutant zebrafish result in altered mitochondrial morphology compared to wild-type siblings (Chapman et al., 2013). The aspect ratio of mitochondria (major axis of bounding ellipse / the minor axis) in homozygous mutant neurons (1.75 ± 0.54) is significantly reduced in comparison to wild type neurons (2.12 ± 0.91; Chapman et al., 2013). Homozygous MFN2^L285X/L285X^ zebrafish have normal CNS development and swimming behavior with smaller body sizes than wild type siblings. The survival rate gradually declines after 175 days in homozygous mutant fish (Chapman et al., 2013), suggesting an adult onset of disease. Additionally, adult homozygous MFN2^L285X/L285X^ mutant zebrafish showed a characteristic droopy tail feature, frequently swimming at an angle of more than 30 degrees below horizontal, further demonstrating motor defects. A potential mechanism of the motor defect was revealed by the analysis of NMJ pathology. Adult homozygous MFN2^L285X/L285X^ mutant zebrafish displayed a reduced area of the pre- and post-synaptic compartments marked by SV2 and α-bungarotoxin, respectively, (Chapman et al., 2013). Time-lapse tracking of mitochondrial movements *via* Mitotracker in cultured neurons revealed a selective inhibition of retrograde motion in MFN2^L285X/L285X^ mutant neurons compared to wild type. Furthermore, the quantification revealed a reduced velocity of transport in the retrograde direction along with a reduction in the number of motile mitochondria. The altered mitochondrial dynamics could account for the NMJ pathology and axonal degeneration in CMT type 2A patients.

Charcot–Marie-Tooth2b (CMT2b) is a subtype of CMT2 axonal form with an early onset that preferentially affects sensory neurons (Rotthier et al., 2009). CMT2b is an autosomal dominant disease caused by five missense mutations in the Rab7 gene (L129F, K157N, N161I, N161T, and V162M; Verhoeven et al., 2003; Houlden et al., 2004; Meggouh et al., 2006; Wang et al., 2014). Rab7 is a small GTPase responsible for converting early endosomes to late endosomes, biogenesis of lysosomes, and maturation of autophagosomes (Bucci et al., 2000; Hyttinen et al., 2013). The amino acid substitutions linked with CMT2b occur in the proximity of the GTP-binding pocket and hydrolysis domains and result in continuous activation of Rab7 GTP bound form (De Luca et al., 2008; Spinosa et al., 2008; McCray et al., 2010). The zebrafish Rab7 protein is 97.6% identical to the human protein sequence, including amino acid residues that are affected in human disease (L129F, K157N, N161T, V162M; Verhoeven et al., 2003; Houlden et al., 2004; Meggouh et al., 2006). All these mutations were generated in zebrafish *rab7* cDNA constructs (-*3.1ngn:GFP-Rab7-cmt2b*) using single site mutagenesis and were expressed in zebrafish Rohon-Beard (RB) spinal sensory neurons (Ponomareva et al., 2016). RB neurons have a stereotyped morphology, which is an extension of two central axons that ascend and descend ipsilaterally in the spinal cord and an extension of a third peripheral axon to the skin where it branches extensively. In the developing period of RB neurons at 23hpf, the CMT2b Rab7 mutants exhibited altered morphology. CMT2b K157N and V162M mutants showed defects in peripheral axon length and all CMT2b mutants showed decreased branching of peripheral axons (Ponomareva et al., 2016). To determine whether there was a gain of function or partial loss of function of Rab7, a constitutively active Rab7 mutant zebrafish was generated. Its RB neurons phenocopied the morphological characteristics of RB neurons of CMT2b Rab7 mutant zebrafish, indicating a gain of function of Rab7 in CMT type 2b (Ponomareva et al., 2016). Additionally, high-speed, high-resolution *in vivo* imaging of endosome movement using swept field confocal microscopy revealed decreased vesicle speed in the K157N Rab7 mutant zebrafish.

Recently whole-exome sequencing of a Korean family with axonal CMT revealed a *de novo* missense mutation (p.Y223H) in the diacylglycerol O-acyltransferase 2 (DGAT2) gene (Hong et al., 2016). DGAT2 encodes an endoplasmic reticulum-mitochondria-associated membrane protein, acyl-CoA:diacylglycerol acyltransferase, which is involved in the triacylglycerol biosynthesis pathway. Overexpression of the mutant human DGAT2 gene in zebrafish results in the abrogated formation of neuronal fascicles with intact muscle fibers at 3 dpf, which is one characteristic phenotype of CMT2 (Hong et al., 2016).

CMT is associated with over 80 genetic mutations, including a large gene family of aminoacyl-tRNA synthetases (ARS; Meyer-Schuman and Antonellis, 2017; Wei et al., 2019). ARS catalyzes the attachment of amino acids to their matching tRNAs, which is a crucial step in protein synthesis (Ibba and Söll, 2001). Overexpression of CMT-ARS variants in *drosophila* impairs global cellular translation in neurons (Niehues et al., 2015). Additionally, overexpression of human CMT- histidyl-tRNA synthetase (HARS1) mutants in zebrafish embryos resulted in short sensory and motor neuron axons with misguided ventral projections (Mullen et al., 2021). Furthermore, the mutant fish displayed an altered motor phenotype with a twitch-like swimming pattern. Investigation of Cycloheximide (a protein synthesis inhibitor) on zebrafish embryos resulted in shorter DRG axon length and disrupted neurite morphology, suggesting a role for ARS-linked protein synthesis inhibition in CMT (Mullen et al., 2021). The generation of different zebrafish models mimicking CMT (Table 2) has helped in understanding the pathology of the disease which may be further used to derive potential therapeutic interventions.

**Table 2:** Zebrafish CMT models and their distinct phenotypes.

Mutated CMT genes modeled in zebrafish	Characteristic phenotypes	Method used to create the model
*MNF2*	• Significant reduction in aspect ratio of mitochondria	T > A mutation in exon8 of zebrafish *MFN2* gene
	• Small body size	
	• Droopy tail	
	• Altered neuromuscular junction morphology	
	• Inhibition of mitochondrial retrograde movement	
	• Reduced number of motile mitochondria	
*Rab7*	• Altered Rohon-Beard sensory neuron morphology	Single site mutagenesis in zebrafish *rab7* cDNA constructs (-3.1ngn:GFP-Rab7-cmt2b) causing four missense mutations in the Rab7 gene (L129F, K157N, N161T, V162M)
	• Defects in peripheral axon length	
	• Decreased peripheral axon branching	
	• Reduced endosomal vesicle speed	
*DGAT2*	• Abrogated formation of neuronal fascicles	Overexpression of the mutant human DGAT2 gene in zebrafish
*HARS1*	• Short sensory and motor axons	Overexpression of human CMT- histidyl-tRNA synthetase (HARS1) mutants in zebrafish
	• Misguided ventral projections of sensory and motor neurons	
	• Altered motor phenotype	

## Amyotrophic lateral sclerosis

**Figure 3:**
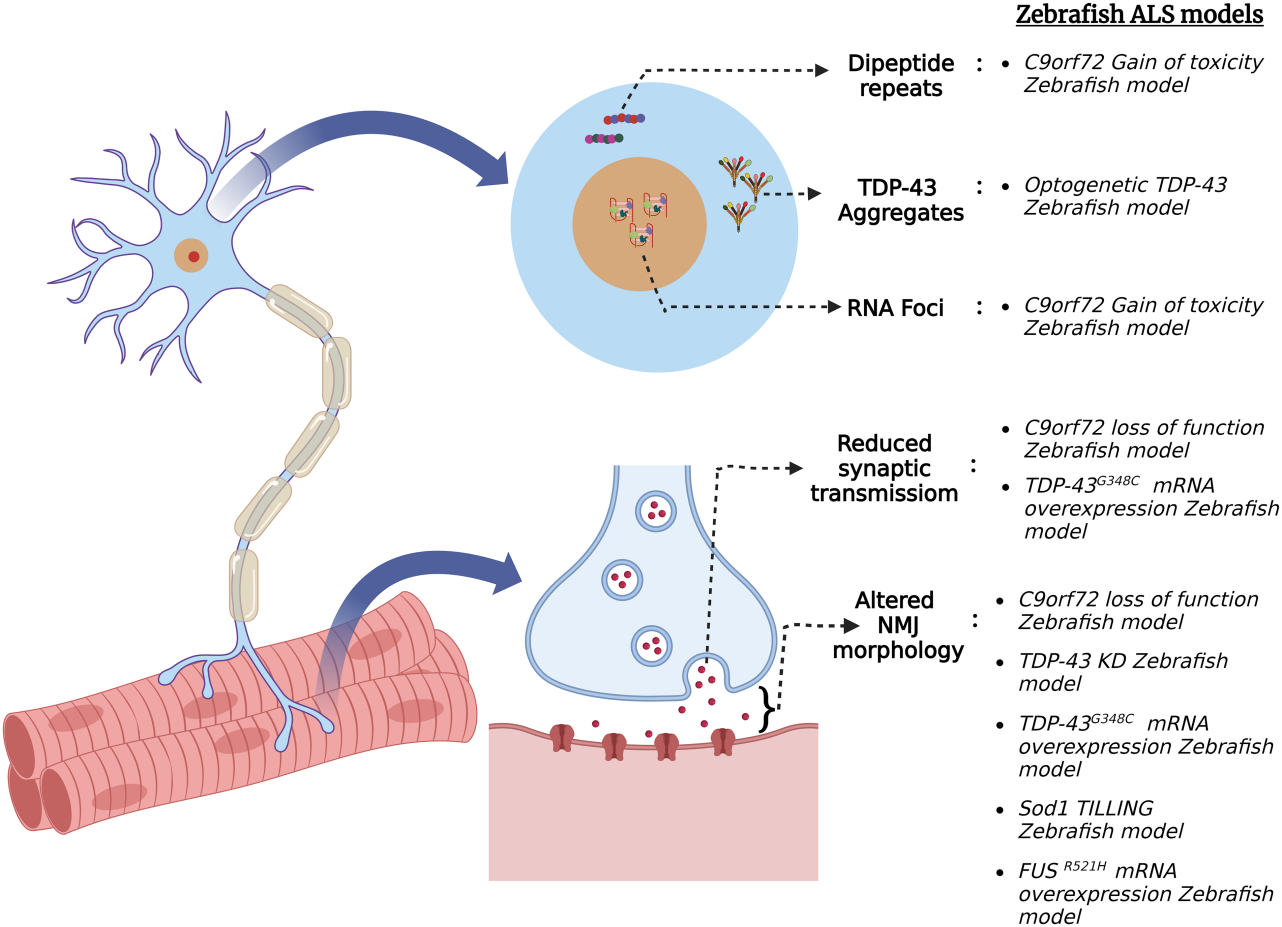
Schematic summary of various pathological signatures of ALS that are replicated in zebrafish ALS models. Zebrafish models were generated *via* several genetic approaches for the four major ALS genes (C9orf72, *FUS*, *TARDBP* (TDP-43) and *SOD1*) and they exhibit ALS hallmarks such as formation of toxic dipeptide repeats, TDP-43 aggregation, RNA foci formation, altered neuromuscular junction (NMJ) morphology and reduced synaptic transmission at NMJ.

Amyotrophic lateral sclerosis (ALS) is a fatal neurodegenerative disorder that results in the selective loss of motor neurons. ALS typically begins in middle age as focal muscle weakness, quickly progressing to generalized muscle wasting and death within 2–5 years of clinical onset. In 1869 Jean-Martin Charcot formally defined ALS, although the earliest cases were described by Aran and Cruveilhier in 1848 and 1853, respectively. Spinal onset is most commonly seen in ALS and primarily affects the limbs, but some cases report bulbar onset involving the mouth, jaw, tongue, and facial muscles. Riluzole, Edaravone and AMX0035 (sodium phenylbutyrate and taurursodiol) are currently the only FDA-approved drugs used to prolong the life expectancy of patients but do not reverse the progression of the disease. Approximately 10% of ALS cases are recorded as familial (fALS) and 90% are thought to be sporadic (sALS). Due to the incomplete penetrance of the disease and the unavailability of genetic data for ALS, some familial cases can be recorded as sporadic. ALS has been linked to over 40 genetic mutations (Kuźma-Kozakiewicz and Kwieciński, 2009). The most common genetic mutation linked to ALS is the hexanucleotide repeat expansion (HRE) GGGGCC (G4C2) present in the first intronic region of the C9*orf*72 gene, seen in 40% of fALS cases (DeJesus-Hernandez et al., 2011; Renton et al., 2014). Other genetic mutations linked to ALS are *SOD1,* seen in 20% of cases (Rosen et al., 1993) and *TARDBP* and *FUS*, observed in <5% of cases (Sreedharan et al., 2008; Vance et al., 2009). *SOD1* was the first gene to be linked with the onset of ALS in 1993. TDP-43 cytoplasmic inclusions in neuronal as well as non-neuronal cells are considered one of the characteristic hallmarks of ALS, and are found in almost 97% of ALS cases. The functioning of various cell-intrinsic pathways, such as mitochondrial bioenergetics, nuclear-cytoplasmic transport, RNA metabolism, and axonal transport, is found to be disturbed in ALS, which further causes motor neuron degeneration (Vance et al., 2009; Liao et al., 2019; Mehta et al., 2021). But the factor triggering the selective degeneration of motor neurons remains elusive. To answer this question, different zebrafish models have been generated which display the pathological hallmarks of ALS seen in humans (Figure 3).

Mutations of the *C9orf72* gene have been implicated in both gain-of-toxicity and loss-of-function mechanisms. To that end, zebrafish models have been generated to study both pathological mechanisms in C9orf72 ALS. For instance, a stable zebrafish gain-of-function model expressing 89 G4C2 HRE exhibited the hallmark characteristics of C9orf72 ALS such as dipeptide repeats (DPR) and RNA foci formation (Shaw et al., 2018). Additionally, the fish displayed motor neuron loss, muscle atrophy, motor impairment, cognitive abnormalities, and reduced adult survival, supporting the gain of toxicity mechanism. On the other hand, a stable *C9orf72* (C9-mir) zebrafish loss-of-function model was created *via* a targeted miRNA gene silencing approach. The endogenous zebrafish *C9orf72* gene expression was specifically and ubiquitously knocked down using mi-RNAs targeting the 3′-UTR region of the gene (Butti et al., 2021). C9-miR fishes showed early motor defects of reduced swimming distance and velocity and motor neuron degeneration at 6 dpf. TDP-43 clusters were observed in the skeletal muscles of the adult C9-miR fishes, which is a hallmark feature of ALS observed in ~97% of the cases. Additionally, the C9-miR fishes displayed muscular atrophy with a significant reduction in fiber thickness, reduced frequency of mEPCs and impaired release of quantal synaptic vesicles (Butti et al., 2021). Thus, this *C9orf72* zebrafish loss-of-function model replicates the hallmarks of ALS and can be further used to study the pathogenesis of the disease.

RAN-translation of *C9orf72* hexanucleotide repeat sequences in both sense and antisense directions generates five species of DPRs, i.e., poly-glycine–alanine (GA), poly-glycine-proline (GP), poly-glycine-arginine (GR), poly-proline-arginine (PR), poly-proline-alanine (PA), and poly-proline-glycine (PG; Mori et al., 2013). These DPRs are found in post-mortem brain tissues, some spinal tissues and skeletal muscles of patients suffering from ALS (MacKenzie et al., 2013; Gomez-Deza et al., 2015; Cykowski et al., 2019). Out of these five DPR species, the arginine-containing ones are known to be more toxic in cells and animal models (Mizielinska et al., 2014; Wen et al., 2014; Swaminathan et al., 2018). A zebrafish model was created to mimic the GR toxicity and to screen for its potential suppressors. Zebrafish embryos were injected with RNA encoding ATG-mediated codon-optimized 100 × GR which increased apoptotic cell death in 1–4 dpf zebrafish brain (Riemslagh et al., 2021). Additionally, the analysis of NMJ morphology in the trunk by SV2 and α-BTX staining revealed a reduction in axonal protrusions of the motor neuron axonal structure. Trolox, an antioxidant, rescued the apoptotic phenotype of the GR-injected fish as it inhibited the formation of reactive oxygen species (ROS), thereby reducing oxidative stress (Riemslagh et al., 2021).

TDP-43 is an RNA/DNA binding protein encoded by the *TARDBP* gene. It is primarily localized in the nucleus, but due to the presence of nuclear localization and nuclear export signals, it shuttles between the nucleus and cytoplasm (Archbold et al., 2018). TDP-43 regulates pre-mRNA splicing, mRNA stability, mRNA transport and translation (Ling et al., 2013). In addition to being a pathological marker, it is also found to be a causative agent for both sporadic and familial cases of ALS. Morpholino knockdown of *tardbp* in zebrafish (TDP-43 KD) resulted in shorter swim duration, distance, and velocity (Campanari et al., 2021). Additionally, staining of the pre- and post-synaptic puncta with α-BTX and SV2, respectively, revealed a reduction in post-synaptic AchR. Since AchE is essential for NMJ integrity, AchE activity was assessed *via* Ellman assay spectrophotometry on total fish protein extract in TDP-43 KD zebrafish. TDP-43 KD embryos present a significant reduction in AchE activity which can be rescued by the injection of human *TARDBP* mRNA (Campanari et al., 2021). Therefore, TDP-43 exhibits a functional link with AchE activity in ALS pathogenesis.

Cytoplasmic mislocalization of TDP-43 is one of the characteristic signatures in most ALS patients. A zebrafish model has been developed using an optogenetic approach in which external light illumination can cause TDP-43 to mislocalize and form aggregates in the cytoplasm (Asakawa et al., 2020). An optogenetic TDP-43 variant, i.e., mRFP1-tardbp-CRY2olig (opTDP-43z), having light dependent oligomerization module of cytochrome-2 attached to an intrinsically disordered region (IDR) of TDP-43 was generated and expressed in zebrafish. This opTDP-43z was expressed in spinal motor neurons and sensory Rohon-Beard Cells of zebrafish using UAS:GAL4 system. After 90 min of blue light illumination, opTDP-43z mislocalization was observed in both type of cells. Specific expression of opTDP-43z in caudal primary motor neurons and 3-h long illumination by blue light at stage 28–31 hpf results in cytoplasmic mislocalization of opTDP-43z and reduced axon length but no aggregates of TDP-43 were observed (Asakawa et al., 2020). Long-term illumination of blue light (24–48 h) results in cytoplasmic mislocalization and the formation of TDP-43 aggregates in zebrafish spinal motor neurons at 72hpf. An altered variant of opTDP-43z bearing an ALS mutation A315T in the IDR region was generated (opTDP-43h^A315T^) and expressed in zebrafish spinal motor neurons (Asakawa et al., 2020). Within 24 h of blue light illumination, cytoplasmic mislocalization and the formation of opTDP-43h^A315T^ aggregates was observed, along with the seeding of intrinsic TDP-43 to mislocalize and form aggregates. Almost 13% of illuminated zebrafish at expressing opTDP-43h^A315T^ failed to inflate swim bladder at 5 dpf and showed locomotor defects (Asakawa et al., 2020). Therefore, this zebrafish model allows for studying the effect of TDP-43 mislocalization and aggregate formation in live zebrafish motor neurons. Moreover, TDP-43 zebrafish models have proven to be efficient in screening potential compounds that may alleviate the symptoms of ALS. As motor neuron degeneration occurs at the terminal stage of the illness, a neuroprotective drug called pimozide was tested on zebrafish. Treatment with 1 μM pimozide on mTDP-43 zebrafish model increased swimming duration, maximum swim velocity, and improved axonal and NMJ phenotypes (Patten et al., 2017). These results were further replicated in FUS and SOD1 zebrafish models. This drug was translated into clinical trials showing improved motor stability in patients, thus proving the potential of zebrafish as a model organism for screening drugs that may reduce or reverse the progression of ALS (Patten et al., 2017). A mutant TDP-43 zebrafish model (mTDP-43) generated by expressing ALS causing mutation G348C of human TARDBP showed motor behavior defects assessed by the touch-evoked escape response (Armstrong and Drapeau, 2013a). Additionally, paired CaP motoneuron—fast-twitch muscle recordings revealed reduced synaptic transmission success rate and reduced end plate current in mTDP-43 zebrafish. Analysis of NMJ morphology in mTDP-43 zebrafish by SV2 (pre-synaptic) and α-bungarotoxin (αBTX; post-synaptic) staining showed an increase in orphaned αBTX clusters and SV2 puncta when compared with wild-type larvae and larvae expressing wild type-TARDBP (Armstrong and Drapeau, 2013a). Chronic (12 h) exposure of mTDP-43 zebrafish larvae to Ca2+ channel agonists as 0.1 μm FPL 64176 or 1 μm Bay K 8644 restored motor defects as swim distance and maximum swim velocity (Armstrong and Drapeau, 2013a). In addition, chronic exposure of mTDP-43 zebrafish larvae to 0.1 μm FPL 64176 or 1 μm Bay K 8644 restored synaptic transmission defects and NMJ structure, proving the scope of Ca^2+^ channel agonists for future ALS therapeutics.

SOD1 is located in the cytoplasm and the intermembrane space of mitochondria. It catalyzes the production of oxygen and hydrogen peroxide from superoxide species and thus acts as an antioxidant (Mccords and Fridovich, 1969). One hundred eighty variants of this gene have been identified in the disease progression and onset. It has been seen that different variants manifest different clinical signatures and symptoms, with some being aggressive (A4V) forms and others related to the slow progression of the disease (D90A; Yamashita and Ando, 2015). SOD1 aggregation has also been linked to protein disulfide isomerase (PDI), which is a chaperone protein and a regulator of redox activity. Pharmacological inhibition of the catalytic activity of PDI results in SOD1 aggregates (Atkin et al., 2006). Using ENU mutagenesis and targeting induced local lesions in genomes (TILLING), missense mutation T70I was generated in the zebrafish *sod1* gene (Da Costa et al., 2014). Protein quantification in 72hpf fish indicated a normal Sod1 expression level but reduced enzymatic activity in T70I fish compared to their control siblings. Mutant T70I fish exhibited increased oxidative stress and altered NMJ phenotype. Furthermore, motor behavior analysis on T70I fish revealed increased resting phase time and decreased burst phase time (Da Costa et al., 2014). This *sod1* zebrafish model displays hallmark phenotypes seen in ALS cases and can be further used to screen potential drugs.

High-throughput screening of 3,768 compounds on mutant TDP-43 (*TARDBP*A315T) *C. elegans* generated 11 positive hits that improved the motility of mutant TDP-43 worms (Bose et al., 2019). These 11 compounds were further tested on zebrafish models with a gain of function mutation in TDP-43 (TARDBP ^G348C^) and SOD1 (SOD-1^G93A^). Among these 11 compounds, TRVA 242 was identified to improve motor function, such as the coiling frequency of embryos and touch-evoked escape response in mutant TDP-43 and SOD1 zebrafish models. Additionally, TRVA 242 was found to rescue the motor axon lengths to wild-type levels in mutant TDP-43, SOD1, and C9ORF72 ALS zebrafish models (Bose et al., 2019). TRVA 242 restored neurotransmission defects in mutant SOD1 zebrafish and the results were phenocopied on the SOD1^G37R^ ALS mouse model, highlighting the potential of zebrafish for screening potential therapeutic drugs in ALS (Bose et al., 2019).

Fused in sarcoma encodes the RNA-binding protein FUS and is associated with the early and juvenile onset of ALS (Zou et al., 2013; Gromicho et al., 2017). TDP-43 aggregates found in most ALS cases are generally absent in FUS-induced ALS, which is instead usually characterized by pathological FUS aggregates. Like TDP-43, FUS plays a role in pre-mRNA splicing, translation, and RNA transport. Almost 50 variants of this gene have been associated with ALS, the majority being missense mutations (Lattante et al., 2013). A zebrafish model characterizing the missense mutation R521H as seen in human FUS-induced ALS was generated. Both mutant R521H fish and morpholino *fus* knockdown zebrafish showed locomotor defects and abnormal motor axon projections at 48 hpf (Kabashi et al., 2011). Whole-cell recordings of CaP motoneurons in larvae expressing the human *FUS*^R521H^ missense mutation (mut*FUS*) showed reduced rheobase current compared with other experimental groups (Armstrong and Drapeau, 2013b). Furthermore, paired CaP motor neuron–fast-twitch muscle cell patch-clamp recordings displayed reduced amplitude of endplate currents (EPCs) and absence of post-stimulation release in mut*FUS* larvae. Additionally, the analysis of NMJ morphology in *mut*FUS larvae revealed an increase in orphaned pre- and post-synaptic terminals (Armstrong and Drapeau, 2013b). This *mut*FUS zebrafish model reveals the pre-clinal synaptic changes that occur prior to motor neuron degeneration and can be further used to track the pathogenesis of the disease.

## Myasthenia gravis

Myasthenia gravis (MG) is a chronic autoimmune disease that causes weakness in the skeletal muscles. The antibodies produced by the body bind, alter or degrade the acetylcholine receptors (AChR) on the muscle fibers, thereby hindering muscle contraction by the motor neurons. An acetylcholinesterase (AChE) inhibitor, pyridostigmine bromide (PB), slows down the hydrolysis of acetylcholine at the synaptic cleft, thereby improving neuromuscular transmission. As PB is a carbamate acetylcholinesterase inhibitor, its activity can be reversed by the decarbamylation of AChE (Harris et al., 1987) and the molecule becomes incapable of AChE inhibition. A zebrafish model was developed for rapid toxicological screening and mechanistic investigation of PB carbamate AChE inhibitor. PB-treated (100 mM) 5 dpf zebrafish larvae showed abnormal pigment deposition, high mortality rate, and significant edema (Fischer et al., 2015). These phenotypes were reduced at 50 mM and 10 mM concentrations of PB. Additionally, 15 mM of PB was found to inhibit 50% of AChE activity. Thus, this zebrafish model can be further used to test more carbamate AChE inhibitors that would alleviate the symptoms of MG.

The reduced density of AChRs on the post-synaptic membrane has been reported in MG. A zebrafish transgenic line was developed to freely control the timing of AChR expression in an AChR-less fish background using a chemical inducible system (Mott et al., 2017). The induction of AChR expression after 2 dpf in an AChR-less zebrafish line showed an improvement in motor behaviors such as swimming distance and the angle of turn which was analyzed at 5 dpf. The AChR-less zebrafish after the induction of AChR expression after 2 dpf was called as *delayed rescue sop* zebrafish. Moreover, the examination of synapse forming sequence between nerve terminals and AChRs was found to be reversed in *delayed rescue sop* zebrafish (Mott et al., 2017). In contrast to the normal synapse-forming sequence where AChR forms before the entry of nerve terminals, the newly formed AChRs followed the guidance of motoneurons in *delayed rescue sop* zebrafish. The analysis of synaptic physiology revealed the absence of mEPCs in muscle cells while no difference was observed in evoked synaptic currents in *delayed rescue sop* zebrafish suggesting a role of spontaneous vesicle release in synaptic dysfunction (Mott et al., 2017). The reduced density of AChRs on the post-synaptic membrane in MG is reported to be compensated by the increase in quantal content which offsets the reduction of AChRs (Plomp et al., 1995). Reduced spontaneous vesicle release has also been seen in MG (Plomp et al., 1995). Studying the physiological role of reduced spontaneous vesicle release in *delayed rescue sop* zebrafish could aid in understanding the physiology of synaptic dysfunction MG.

## Other NMJ disorders

Apart from the most common neuromuscular disorders described in the above sections, a few rare forms of NMJ disorders like Slow channel syndrome (SCS), and Escobar syndrome (ES) have also been modeled in zebrafish. SCS and ES are two forms of congenital myasthenia resulting from mutations in post-synaptic AChRs at the NMJ. SCS is an autosomal dominant post-synaptic disorder caused due to mutation in genes encoding alpha, beta, delta, or epsilon subunits of the AChR. These mutations result in the prolonged opening of the channel, causing prolonged synaptic currents and resulting in a depolarization block (Engel et al., 2015). Escobar syndrome is an autosomal recessive disorder caused by the mutation in the post-synaptic gamma subunit of the AChR. It is characterized by excessive webbing (pterygia), congenital contractures (arthrogryposis), and scoliosis (Escobar et al., 1978; Rajab et al., 2005). Improvement in neuromuscular transmission is seen in Escobar syndrome during development which is thought to be rescued by the presence of the epsilon subunit of the AChR during development (Hoffmann et al., 2006). An intersectional zebrafish model of SCS and ES called *twister* has been generated, which exhibits gain of function characteristics of SCS but shows developmental improvement seen in ES cases. Swimming analysis of the *twi*^+/−^ zebrafish model showed initial disturbances at 48hpf with a prolonged C-bend of body curvature after a mechanical stimulus, and impeded rhythmic swimming (Walogorsky et al., 2012). These defects were rescued at the later developmental stage of 120 hpf and were completely indistinguishable from wild type at 192hpf. Furthermore, whole-cell voltage-clamp recordings from *twi*^+/−^ fast muscles revealed prolonged spontaneous synaptic currents compared with wild-type fish. The decay of these currents accelerated during the development in *twi*^+/−,^ resulting in behavioral recovery. Thus, this intersectional zebrafish model is a valuable tool for investigating the subunit switch of AChR during development and tracking the pathogenesis of both SCS and ES.

## Conclusion

Studies discussed in this review establish that the zebrafish is a powerful model organism to study the genetics and pathogenesis of human NMDs. The structural and molecular similarity of the zebrafish neuromuscular system to humans makes it a valuable model organism to mimic most human NMDs (Babin et al., 2014). Ease of live imaging in addition to high throughput screening of small molecules, give it an advantage over rodent NMD models. As outlined in the above studies, zebrafish have played a beneficial role in discovering drugs that translated into clinical trials and have significantly improved the symptoms in many patients suffering from NMDs (Patten et al., 2017). The ease of applying innovative genetic manipulative tools on zebrafish provides a step up to cope with the continuous discovery of new genetic mutations in most NMDs. Optogenetic approaches to modulate subcellular expression and structure of molecules *in vivo* in zebrafish larvae can lead to the discovery of novel pathways, mechanistic links and new therapeutic interventions. New zebrafish models are continuously developed to study the development, progression and severity of common and rare NMDs, which can be further used for targeted gene therapy and drug discovery.

## Author contributions

SP and JS conceived the work and prepared the final version of the manuscript. JS drafted and prepared this review article and designed the figures. All authors contributed to the article and approved the submitted version.

## Funding

SP is supported by the Canadian Institutes of Health Research (CIHR), and is a FRQS Junior 2 research scholar. He also holds the Anna Sforza Djoukhadjian Research Chair in ALS.

## Conflict of interest

The authors declare that the research was conducted in the absence of any commercial or financial relationships that could be construed as a potential conflict of interest.

## Publisher’s note

All claims expressed in this article are solely those of the authors and do not necessarily represent those of their affiliated organizations, or those of the publisher, the editors and the reviewers. Any product that may be evaluated in this article, or claim that may be made by its manufacturer, is not guaranteed or endorsed by the publisher.
